# Olink Platform-Based Identification of Peripheral Blood Biomarkers for irAEs in Advanced NSCLC

**DOI:** 10.3390/curroncol33100605

**Published:** 2026-10-09

**Authors:** Zhihan Zhang, Bingchun Chen, Hanmei Jiang, Ziyan Zhang, Jianming Mo, Yanyi Hou, Shuang Liu, Ping Xu

**Affiliations:** 1 Department of Pulmonary and Critical Care Medicine, Peking University Shenzhen Hospital, Shenzhen 518036, China; zhangzhihan1999@stu.wchscu.cn (Z.Z.);; 2 Department of Pulmonary and Critical Care Medicine, Targeted Tracer Research and Development Laboratory, Frontiers Science Center for Disease-Related Molecular Network, State Key Laboratory of Respiratory Health and Multimorbidity, West China Hospital, West China School of Medicine, Sichuan University, Chengdu 610041, China; 3 PKU-Shenzhen Clinical Institute, Shantou University Medical College, Shenzhen 518000, China; 19bcchen@stu.edu.cn (B.C.);; 4 Department of Radiotherapy Oncology, Sun Yat-Sen Memorial Hospital, Sun Yat-Sen University, Guangzhou 528406, China; 5 Department of Radiation Oncology, Sichuan Cancer Hospital and Institute, University of Electronic Science and Technology of China, Chengdu 610041, China; 6 Health Science Center, Shenzhen University, Shenzhen 518060, China

**Keywords:** non-small cell lung cancer, immune-related adverse events, biomarkers, Olink profiling

## Abstract

Lung cancer is the leading cause of cancer-related mortality worldwide. Immunotherapy has transformed the treatment landscape for advanced non-small cell lung cancer (NSCLC). However, immune-related adverse events (irAEs) represent a major clinical challenge during immunotherapy. These events can affect any organ system, and severe irAEs may necessitate treatment interruption or lead to life-threatening outcomes. We prospectively enrolled 84 advanced NSCLC patients treated with immunotherapy (June 2022–June 2024, followed until February 2025), including 57 who developed grade 1–4 irAEs and 27 who did not. Peripheral blood was collected from 39 patients at baseline for Olink platform-based proteomic screening, complemented by flow cytometric analysis of lymphocyte subsets. Olink profiling revealed IL13 and TRAIL as the most significantly differentially expressed proteins between irAEs and non-irAE groups. These proteins were enriched in biological processes such as negative regulation of lung ciliated cell differentiation and positive regulation of lung goblet cell differentiation, as well as in asthma and inflammatory bowel disease pathways. Moreover, dynamic changes in total T lymphocytes, T helper cells, NK cells, and the CD4/CD8 ratio were strongly associated with irAE occurrence. These markers, readily measurable in peripheral blood, offer potential for early risk stratification and real-time monitoring.

## Simple Summary

Immunotherapy for advanced NSCLC is hampered by irAEs and the lack of validated biomarkers. We performed Olink-based plasma proteomics and lymphocyte subset analysis on baseline samples. Differential proteins were enriched via GO/KEGG, and LASSO/logistic and Cox regressions were applied. Olink identified IL13 and TRAIL as significantly differentially expressed proteins, enriched in lung ciliated/goblet cell differentiation and asthma/IBD pathways. Dynamic changes in total T, helper T, NK cells, and the CD4/CD8 ratio were strongly correlated with irAE occurrence. Collectively, IL13, TRAIL, and T/NK dynamics emerge as promising peripheral blood biomarkers for early risk stratification and timely intervention.

## 1. Introduction

Lung cancer is one of the cancers with high incidence and mortality rates [1]. Current cancer immunotherapy targets immune checkpoint molecules, revitalizing various immune cells to re-recognize tumor cells, bringing the possibility of long-term survival for patients with metastatic or advanced tumors. Its clinical application heralds a new era in cancer treatment [2]. Despite the remarkable clinical success of immunotherapy, the immune activation it triggers also makes patients susceptible to unexpected irAEs during treatment. These irAEs can affect almost any organ system, including the heart, bone marrow, kidneys, bones, pituitary gland, etc., and severe irAEs such as myocarditis and immune-related pneumonitis can be life-threatening.

However, the mechanisms underlying the occurrence and development of irAEs are currently unclear, possibly related to factors such as the disruption of self-tolerance, cross-presentation of shared antigens, and inflammatory activation [3]. Current predictive studies of irAEs are mostly retrospective, with small samples, single centers, and inconsistent results across numerous experiments [4]. Therefore, we need to optimize statistical methods to provide higher-level evidence-based medical evidence for this issue, establish a biomarker combination model to comprehensively predict immunotherapy efficacy and irAEs, to screen potential populations for irAEs early, provide timely warnings for severe irAEs, assist clinical treatment decisions, and recommend precision oncology.

Olink protein detection technology is a high-throughput protein detection method with the advantages of high sensitivity, high specificity, low sample volume requirement, and high throughput. It is based on a proximity extension assay, using a pair of specific antibodies to recognize and bind to the target protein. These antibodies are labeled with partially complementary DNA oligonucleotides, which come close to each other and complement each other. Subsequently, extension is performed to generate a unique “double-stranded DNA barcode” that corresponds to the target protein. After amplification, the generated DNA barcode is quantitatively detected, thereby achieving accurate quantitative analysis of proteins [5]. For irAEs in cancer patients, multiple panels are available for selection, providing more comprehensive coverage and high-precision characterization of changes in the human immune system. Compared to mass spectrometry, Olink targeted detection has better directionality of research results and can cover low-abundance proteins. One study quantified 2941 proteins in 496 Liverpool Lung Project plasma samples using the Olink^®^ Explore3072 platform, and identified differential proteins through guided feature selection for lung cancer prediction modeling and validation, for early identification [6]. A study selected the Olink Target 96 Inflammation panel to detect serum samples from gastric adenocarcinoma patients, and found that serum immunoproteomics (CXCL1 and CXCL5) was associated with the response to preoperative chemotherapy. Combined with RNA sequencing, immunohistochemistry, etc., a pre-treatment serum protein scoring system was established for response prediction [7]. In summary, Olink protein detection technology is a reliable and accurate test for serum and plasma detection in tumor patients undergoing immunotherapy, and it helps to discover irAE protein biomarkers in patients with NSCLC.

This study aimed to explore irAE-related biomarkers based on Olink protein detection technology from multiple dimensions, including demographic characteristics, past medical history, pathological features, organismal inflammatory and immune status, lymphocyte subsets, and plasma protein levels. It also aims to establish and validate a logistic model for predicting irAEs, assisting clinicians in identifying potential populations for irAEs, providing early warning of severe adverse events and promoting precision immunotherapy.

## 2. Methods

### 2.1. Research Subjects

**Figure 1:**
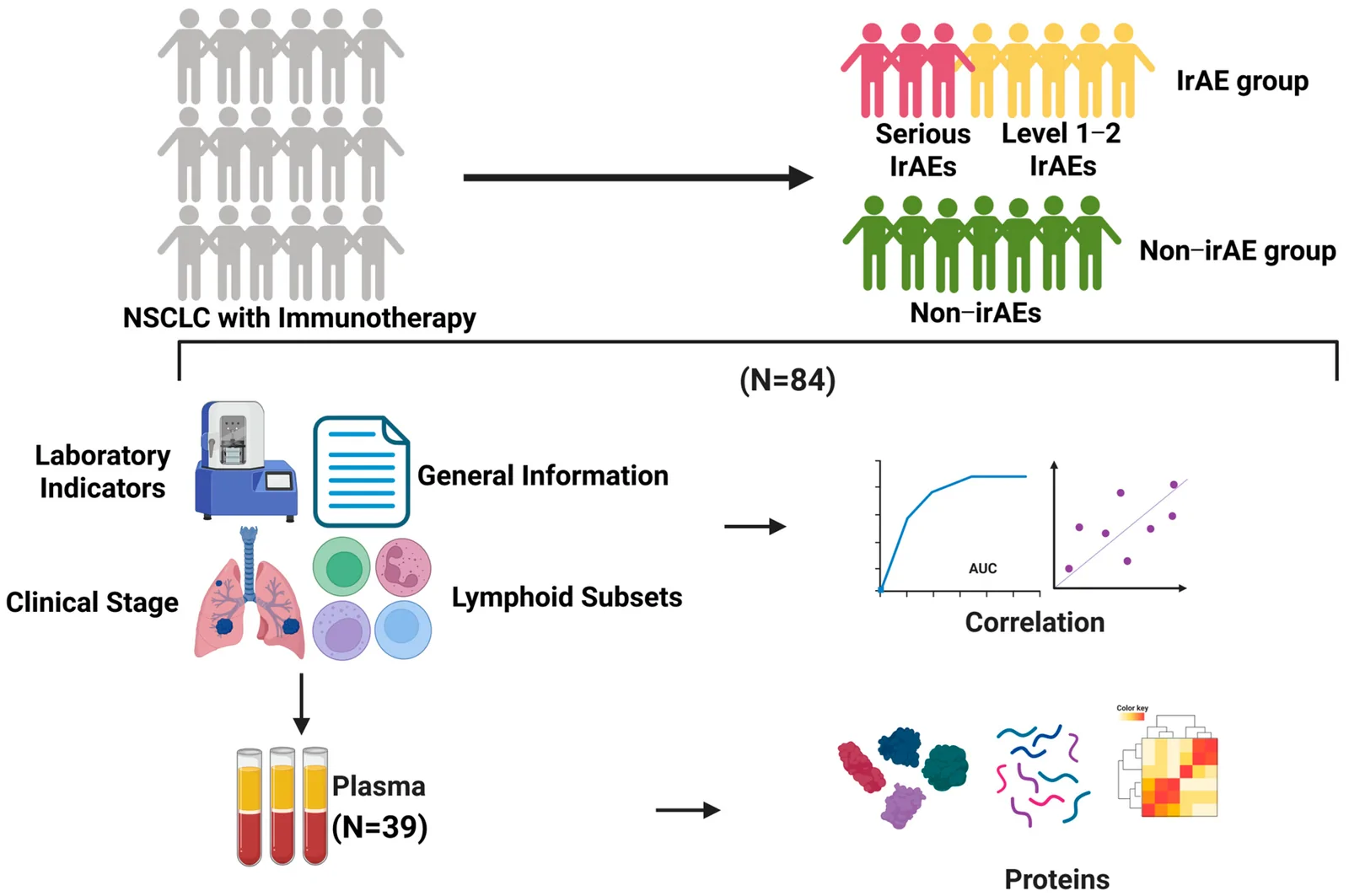
Table of contents image.

We prospectively enrolled patients with advanced NSCLC who received treatment at Peking University Shenzhen Hospital from June 2022 to June 2024, and followed them until February 2025. The inclusion and exclusion criteria are shown in Figure 1. Inclusion criteria: (1) patients with primary lung cancer with clear pathological diagnosis; (2) diagnosed with stage III-IV according to the American Joint Committee on Cancer (AJCC) 8th edition; (3) PD-1/PD-L1 monoclonal antibody alone or in combination; (4) all received PD-1/PD-L1 monoclonal antibody treatment once, and the efficacy could be evaluated; and (5) signed informed consent. Exclusion criteria: (1) pathology contains small cell lung cancer components; (2) occurred or is currently complicated by other malignant tumors within 5 years; (3) those who have been complicated with serious organ damage, serious infection, active autoimmune disease, hematological disease or immunodeficiency before treatment; (4) inability to tolerate immunotherapy; and (5) do not agree to sign the informed consent form. Criteria for withdrawal from the trial: (1) patient death, loss to follow-up; (2) poor compliance, lack of regular treatment and follow-up consultations, resulting in lack of clinical data, imaging and other data; (3) the subject himself requests to withdraw from this study; and (4) other conditions that interfere with the study results in the opinion of the principal investigator, such as adverse events caused by other factors which cannot be ruled out. The variables we included were general information (age, sex, BMI, smoking status, underlying diseases, PS score, etc.), pathological type, clinical stage, laboratory test indicators and composite indicators, peripheral blood plasma proteins, lymphoid subsets, treatment and prognosis, irAE occurrence time, location, and grade. Informed consent was obtained from all participants, and the study was approved by the Ethics Committee of Peking University Shenzhen Hospital.

(1) Laboratory tests indicators: peripheral blood samples were collected from the study population at baseline (within 3 weeks before treatment, assuming that various indicators, immune cell levels, and protein levels were relatively stable during this period, the treatment had little impact, and no irAEs occurred; indicated by “pre”) and after treatment (i.e., the time point when irAEs occurred in the irAE group, and the last blood draw time point after more than 10 cycles in the non-irAE group; indicated by “post”). The change in parameters after treatment minus parameters before treatment is expressed as “dif = post-pre”. Compare the difference between the transient state at the time of irAE onset and the long-term steady state in patients without irAEs.

(2) Single indicators: including absolute peripheral blood leukocyte count, absolute neutrophil count, absolute lymphocyte count, absolute monocyte count, absolute eosinophil count, absolute basophil count, absolute erythrocyte count, hemoglobin level, platelet count, creatinine level, total protein level, albumin level, lactate dehydrogenase level, C-reactive protein (CRP) level, total lymphocyte percentage, T lymphocytes, helper T cell percentage, cytotoxic T cell percentage, helper/cytotoxic T cell ratio, NKT-like cell percentage, B cell percentage, NK cell percentage, absolute total lymphocyte count, absolute T lymphocyte count, absolute helper T cell count, absolute cytotoxic T cell count, absolute NKT-like cell count, absolute B cells, absolute NK cells, etc.

(3) Composite indicators formed by single indicators above: NLR, MLR, ELR, BLR, NLR, PLR, PNI, and SII.

[Explanation] lymphocytes: CD3+ T cells; helper T cell: CD4+ T cell; cytotoxic T cell: CD8+ T cell; helper/cytotoxic T cell ratio: CD4+/CD8+ ratio; NKT-like cell: CD3+CD56+CD16+ natural killer T-like cells; B cell: CD3-CD19+ cell; NK cell: CD3-CD16+CD56+ cell. NLR = neutrophil count/lymphocyte count; MLR = monocyte count/lymphocyte count; ELR = eosinophil count/lymphocyte count; BLR = basophil count/lymphocyte count; PLR = platelet count/lymphocyte count; PNI (prognostic nutritional index) = serum albumin + 5 × lymphocyte count; SII (systemic immune inflammation index) = (platelet count × neutrophil count)/lymphocyte count.

(4) Plasma proteins: baseline (within 3 weeks before treatment) peripheral blood samples were collected, plasma was extracted, and Olink platform protein detection technology was used to screen for differential proteins.

### 2.2. Outcome Definitions and Follow-Up Censoring

The primary endpoint of this study was the occurrence of immune-related adverse events (irAEs), which were classified as severe (grade ≥ 3) or mild (grade 1–2) according to the CSCO Guidelines for the Management of Toxicities Associated with Immune Checkpoint Inhibitor Therapy.

(1) For patients who developed severe irAEs during follow-up, the event time was defined as the date of the first documented severe irAE. For patients who experienced only mild irAEs (grade 1–2) and did not progress to severe irAEs throughout the follow-up period (with at least 10 treatment cycles), the event time was defined as the date of the first mild irAE.
(2) For patients who did not experience any irAE during follow-up (with at least 10 cycles), they were censored at the end of the follow-up period; this censoring time was not considered a study endpoint, but rather the time at which observation ceased for event-free individuals.

### 2.3. Experimental Methods

Peripheral blood plasma protein detection was performed as follows: (1) Blood collection: blood samples (4 mL) were collected from target patients (baseline level) in the fasting state (fasting for at least 6 h) into long purple tubes at room temperature, stored in a 4 °C refrigerator, and centrifuged and aliquoted within 2 h after collection. (2) Processing: the whole blood sample in the long purple tube was centrifuged at 2000 rpm for 10 min, the plasma was aspirated, and frozen at −80 °C, 500 µL per tube. (3) Differential protein detection: experiments on plasma protein extracts were performed in the same batch. Proteins were measured using the Olink^®^ 96 panel (Olink Proteomics AB, Uppsala, Sweden) according to the manufacturer’s instructions. Pairs of oligonucleotide-labeled antibody probes bound to their targeted protein, and if the two probes were brought in close proximity, the oligonucleotides hybridized in a pair-wise manner. The addition of a DNA polymerase led to a proximity-dependent DNA polymerization event, generating a unique PCR target sequence. The resulting DNA sequence was subsequently detected and quantified using a microfluidic real-time PCR instrument (Signature Q100, LC-Bio Technology CO., Ltd., Hangzhou, China). The resulting Ct-data were then quality-controlled and normalized using a set of internal and external controls. The final assay read-out was presented in Normalized Protein eXpression (NPX) values, which is an arbitrary unit on a log2-scale where a high value corresponds to a higher protein expression. The four internal controls were designed to mimic and monitor the different steps of the PEA. They consisted of two non-human proteins with matching antibody probes that functioned as incubation/immuno controls, an IgG antibody with two matching probes attached to it that functioned as an extension control, and a complete double-stranded amplicon that functioned as a detection control. The internal controls were introduced to all samples as well as to the external controls. The external controls consisted of a triplicate of negative controls used to calculate the limit of detection (LOD), as well as a triplicate of interplate controls (IPCs) containing 92 sets of antibodies with both matching probes for each assay attached to them that were used for normalization. For each sample and assay, NPX was calculated by the following equations: 1. Ct(analyte) − Ct(extension control) = dCt(analyte) (to decrease technical variation); 2. dCt(analyte) − Ct(median IPC) = ddCt(analyte) (to improve inter-plate variation); and 3. correction factor(analyte) − ddCt(analyte) = NPX(analyte) (for more intuitive data). The correction factor was a set variable unique for each assay and reagent lot. Quality control of the data was performed in two steps: First, the standard deviation for each of the incubation/immuno controls and the detection control was calculated for each run. A run passed quality control only if the standard deviation for each of the controls was below 0.2. Secondly, each sample was quality-controlled using incubation control 2 and the detection control. The run median of each of the controls was calculated, and all samples within the run were compared to that. Samples that fell more than ±0.3 NPX from the plate median with regard to these two controls failed the quality control and received a QC warning in the data output file. All assay validation data (detection limits, intra- and inter-assay precision data, etc.) are available on the manufacturer’s website (www.olink.com).

### 2.4. Statistical Analysis

All statistical analyses were performed using R software (version 4.5.1; R Foundation for Statistical Computing, Vienna, Austria).

The proportion of missing values is less than 10% (9.2%, 1085/11790). The median and Predictive Mean Matching (PMN) methods were used. Considering the large data deviation after PMN prediction, the median imputation was finally selected. R Studio statistical software (version 4.5.1) was used to analyze baseline data. Continuous variables are expressed as mean ± standard deviation (SD), and inter-group differences were analyzed using *t*-tests. Categorical variables are presented as percentages, and inter-group comparisons were performed using χ2 tests or Fisher’s exact test. A *p*-value of <0.05 was considered statistically significant.

To avoid overfitting and identify the most robust predictors from the high-dimensional multi-omics dataset, we initially employed LASSO (Least Absolute Shrinkage and Selection Operator) regression with L1-penalization. The optimal regularization parameter (λ) was determined via 10-fold cross-validation, minimizing the cross-validated binomial deviance. However, to enhance model parsimony and prevent the inclusion of overly redundant variables, we did not strictly select the λ with the minimum deviance (λ.min). Instead, we applied a more stringent penalty by selecting the largest λ within one standard error of the minimum (λ.1se), ultimately setting the lambda value at 0.082. Variables with non-zero coefficients at this optimal λ were retained as significant predictors.

We extracted these significant predictors along with their respective coefficients (coeff) and subsequently incorporated them into a multivariable logistic regression model to estimate the probability of developing irAEs. The final fitted model was visually presented as a nomogram, enabling individualized risk stratification by mapping each predictor’s value to a corresponding point scale, with the total sum corresponding to the predicted probability of irAEs.

To evaluate the model’s performance, we conducted a comprehensive assessment: Discrimination: the area under the receiver operating characteristic curve (AUC), along with sensitivity and specificity, was calculated to quantify the model’s ability to distinguish between patients with and without irAEs. Calibration: calibration plots were generated to visualize the agreement between the predicted probabilities and the observed frequencies of irAEs. The Hosmer–Lemeshow goodness-of-fit test was further applied, with a *p*-value > 0.05 indicating acceptable calibration. Clinical utility: decision curve analysis (DCA) was performed to evaluate the net clinical benefit across a range of threshold probabilities, comparing the “treat-all” and “treat-none” strategies to assess the practical applicability of our model in clinical decision-making.

Given the limited sample size relative to the number of candidate predictors, rigorous internal validation was conducted to correct for optimism and assess model stability. We employed two complementary resampling strategies: (1) bootstrap resampling with 1000 iterations (R = 1000), where the entire modeling process (including LASSO selection) was repeated in each bootstrap sample to calculate the average optimism, which was then subtracted from the apparent performance to obtain the optimism-corrected estimate; and (2) 10-fold cross-validation, where the dataset was randomly partitioned into 10 equal subsamples, with model performance averaged across all folds. The final validated performance metrics are expressed as mean AUC ± standard deviation (SD).

For the exploratory analysis of factors associated with the time-to-onset of irAEs, we specifically focused on baseline pre-treatment variables. First, we performed a multicollinearity diagnosis using the Variance Inflation Factor (VIF); variables with VIF > 10 were sequentially excluded to mitigate estimation instability. Subsequently, Cox proportional hazard regression was employed to screen for candidate factors associated with the timing of irAE occurrence. To refine this set and avoid overfitting, we applied a stepwise regression algorithm based on the Akaike Information Criterion (AIC) for both backward and forward elimination, selecting the optimal subset of independent predictors. Finally, to explore the direction and magnitude of these associations, we plotted ROC curves for the selected independent variables and calculated their respective Pearson correlation coefficients against the continuous timing variable, quantifying the linear relationships between baseline immunological/inflammatory markers and the earliness of irAE onset.

Box plots and interquartile range (IQR) analysis were performed on internal quality control values in each sample to understand the degree of deviation in internal quality control testing for each sample. The statistical repeatability of each sample was observed and evaluated using heatmaps. Volcano plots were used to observe the distribution of differentially expressed proteins, and the TOP5 differentially expressed proteins with the smallest *p*-values were selected to generate individual ROC curves and calculate the overall ROC curve. Gene Ontology (GO) analysis was performed, including three ontologies: biological process, cellular component, and molecular function, represented by circle plots. In addition, pathway enrichment analysis (Kyoto Encyclopedia of Genes and Genomes, KEGG) [8,9] was performed to identify pathways that were significantly enriched in differentially expressed proteins compared to the entire proteome background, represented by bubble plots.

## 3. Results

### 3.1. General Characteristics of the Research Subjects

**Figure 2:**
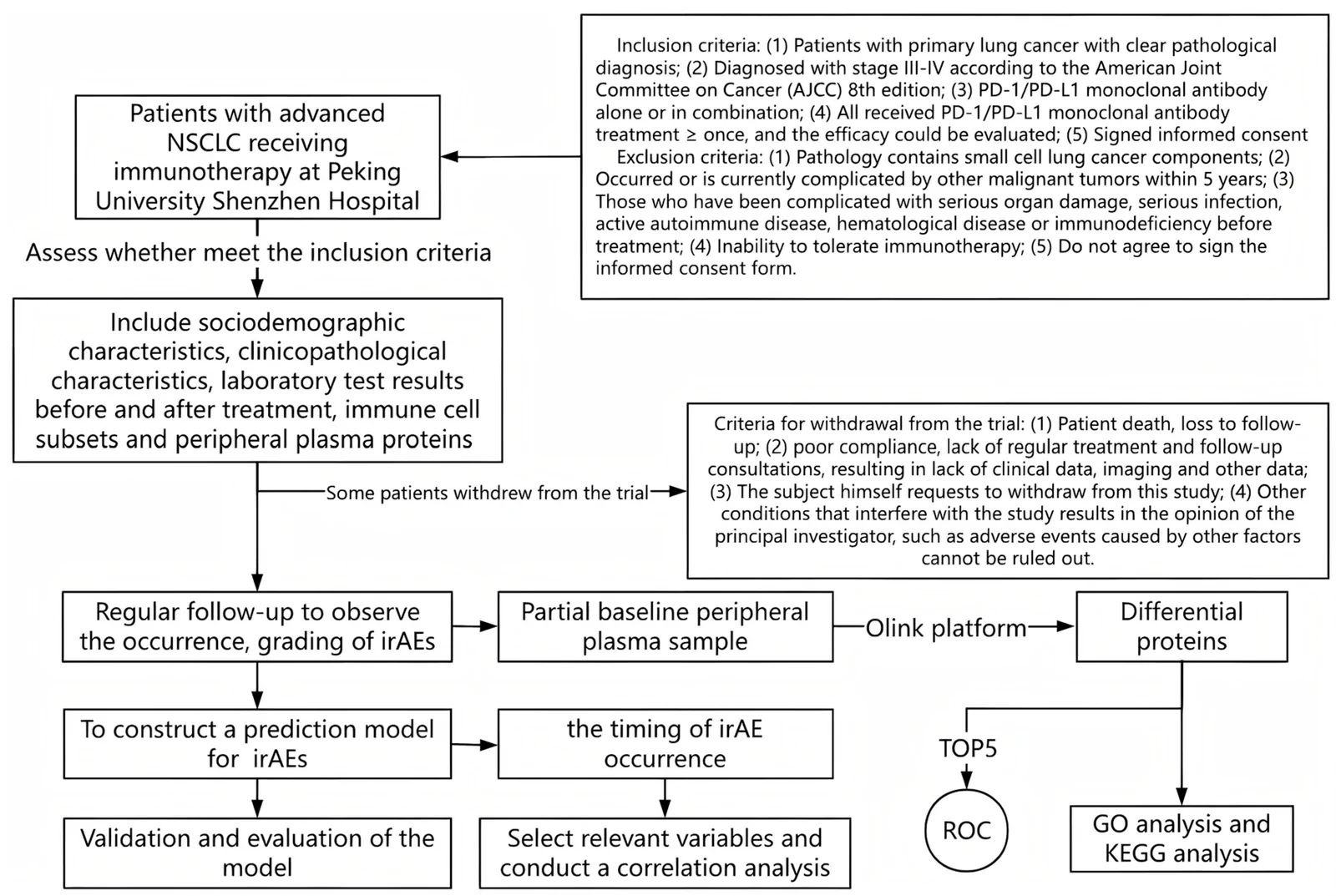
Flow chart.

Based on our inclusion and exclusion criteria, we enrolled 228 patients, of whom 138 withdrew from the trial (Figure 2). A total of 84 cases were finally included after excluding those with incomplete basic and laboratory data. The irAE group was defined as those who had experienced or currently had irAEs, with a total of 57 patients; the non-irAE group was defined as those who had not experienced irAEs until the end of follow-up, with a total of 27 patients (Table 1).

The average age of the irAE group was 67.0 ± 7.5 years and the non-irAE group was 63.8 ± 10.2 years (*p* = 0.11). The proportion of females in the total was 73/84 (86.90%). The irAE group included two patients with severe lung cancer with a PS score of 3–4, and the non-irAE group included one patient with severe lung cancer with a PS score of 3–4 (*p* = 0.39). In addition, there were no statistically significant differences between the irAE group and the non-irAE group in terms of underlying diseases, clinical stage (*p* = 0.84), pathological type (*p* = 0.30), and treatment regimen (*p* = 0.57).

In addition, the incidence rates of irAEs in the skin, endocrine system (thyroid, pituitary, etc.), liver, kidney, lung, gastrointestinal tract, blood system, heart, nervous system, infusion reactions, eye, rheumatic immune system, and other sites in the irAE group were 33.33%, 29.82%, 14.04%, 5.26%, 24.56%, 5.26%, 5.26%, 22.81%, 8.77%, 10.53%, 3.51%, 3.51%, and 8.77% respectively.

### 3.2. A Prediction Model of IrAEs and Related Biomarker

**Figure 3:**
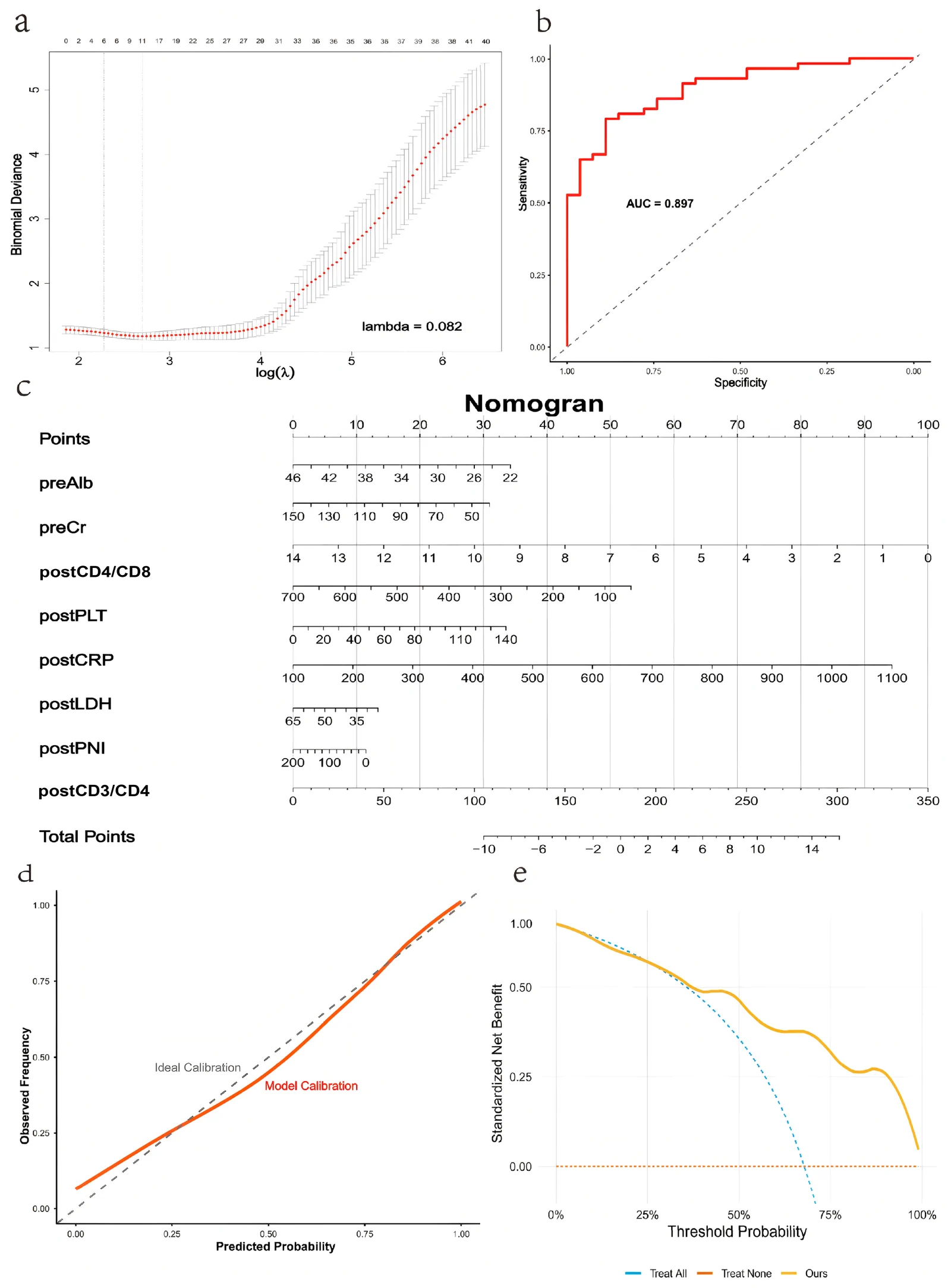
(**a**) Cross-validation plot of irAE prediction model. (**b**) The model’s ROC curve. (**c**) A nomogram of irAE prediction model. (**d**) Calibration curve. (**e**) Decision curve. Note: (**a**) The LASSO analysis uses cross-validation to select the optimal lambda value. Because the penalty was low, the number of variables selected was too large. Therefore, the penalty was increased, and the final lambda value selected was 0.082. (**d**) Calibration curve: the horizontal axis represents the probability predicted by the model, and the vertical axis represents the observed frequency; the dashed line represents the ideal calibration line, and the solid line represents the actual calibration of the model. (**e**) Decision curve: the horizontal axis represents the probability of using the predicted probability as the decision threshold, and the vertical axis represents the standardized net benefit; Our Model represents the standardized net benefit of using our prediction model under different thresholds; All represents the net benefit of taking action regardless of the predicted probability; None represents the net benefit of taking no action regardless of the predicted probability.

Baseline peripheral blood samples were successfully obtained from all 84 enrolled patients. Using existing parameters, we included all the above variables in the dataset of 84 patients. For patients who developed irAEs, we included their baseline data and the data at the time of irAE onset. For patients who did not develop irAEs during the follow-up period (greater than 10 cycles), we included their baseline data and the data at the last follow-up time point. Then, using cross-validation, we selected the optimal lambda (Figure 3a).

After adjustment, we screened out significant predictors, including: pre-treatment albumin value (preAlb), pre-treatment Cr (preCr), pre-treatment CD4/CD8 ratio (preCD4/CD8), post-treatment platelet value (postPLT), post-treatment CRP value (postCRP), post-treatmen LDH (postLDH), post-treatment prognostic nutritional index (postPNI), and post-treatment CD4/CD8 ratio (postCD4/CD8), a total of eight variables (Table 2).

We constructed a logistic regression model to predict the probability of irAEs occurring after treatment with AUC 0.897 (Figure 3b), sensitivity 0.789 and specificity 0.889, indicating that the model had a relatively ideal predictive efficacy. Then we plotted a nomogram (Figure 3c).

We assessed the calibration of the model (Figure 3d). The calibration curve showed good overall agreement between the model-predicted probabilities and the observed frequencies, suggesting that the model was well calibrated without significant systematic bias. Notably, a slight deviation was observed in the intermediate-risk range (predicted probability approximately 0.4–0.6), where the model slightly underestimated the actual risk. This deviation may be attributable to insufficient event density in this range due to the limited sample size.

We further evaluated the clinical net benefit of the prediction model using decision curve analysis (DCA) (Figure 3e), indicating that the model provides good decision support value in clinical practice. The decision curve indicated a net clinical benefit, particularly for intermediate-risk patients.

For internal validation, we used the bootstrap method (1000 resamples) and 10-fold cross-validation to evaluate model stability. The bootstrap method yielded a mean AUC of 0.894 ± 0.038, indicating high discriminative ability with little influence from sampling variability. The 10-fold cross-validation gave a mean AUC of 0.810 ± 0.143; although the value was slightly lower and the standard deviation somewhat larger, this is related to the small sample size (*n* = 84) and random partitioning between folds, yet the overall predictive performance remained acceptable (AUC > 0.80). Both internal validation results consistently demonstrated that the prediction model has good robustness and generalizability potential.

### 3.3. Factors Associated with Severe irAEs

For samples exhibiting severe irAEs (graded 3–4), considering the data imbalance, the model failed to converge even after 10,000 iterations. Therefore, we only used LASSO regression for variable screening. It should be emphasized that these results are exploratory in nature, and the variables identified (Table 3) should be regarded as potential associated factors rather than validated independent predictors.

### 3.4. Correlation Analysis of irAE Occurrence Time

**Figure 4:**
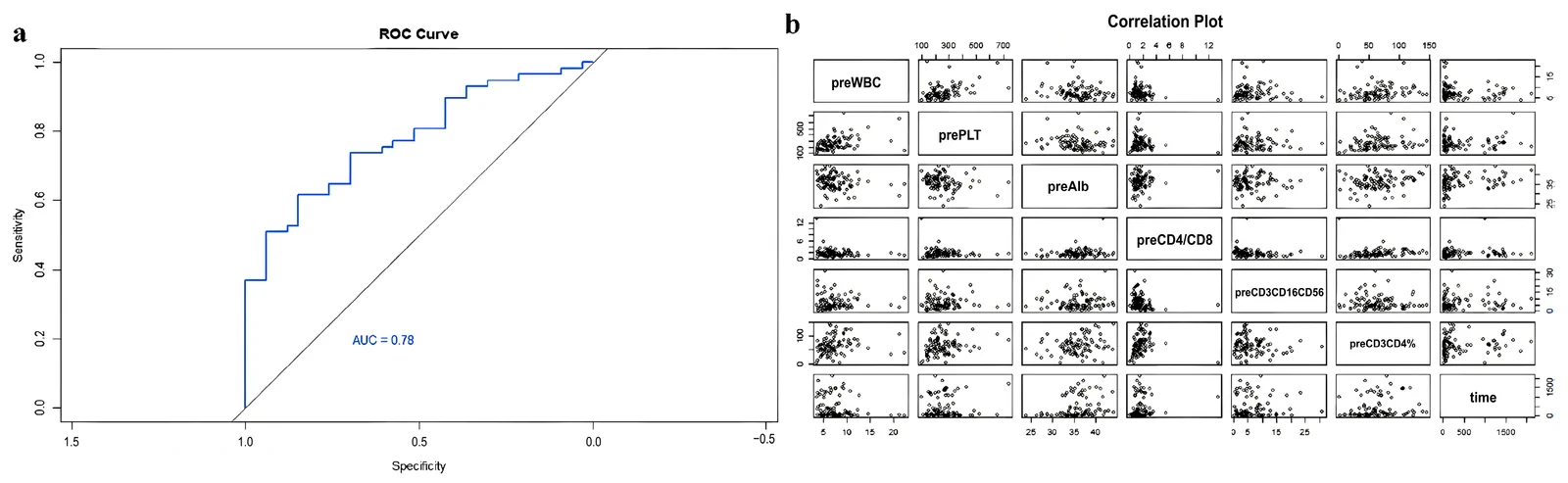
Correlation analysis of irAE occurrence time. (**a**) ROC curve; (**b**) correlation coefficient diagram. Note: the correlation coefficient only measures linear relationships; a high correlation coefficient does not imply causation, but only indicates an association between variables.

Using the timing of irAEs (time from initiation of treatment to the occurrence of irAEs) as the outcome, only pre-treatment variables were included. High VIF variables were excluded. Cox regression was used to screen for factors related to the timing of irAEs. Stepwise regression based on AIC was used for simplification. The screened variables were: pre-treatment WBC (preWBC), pre-treatment platelet count (prePLT), pre-treatment albumin (preAlb), pre-treatment CD4/CD8 ratio (preCD4/CD8), pre-treatment absolute number of NKT-like cells (preCD3CD16CD56), and pre-treatment percentage of T helper cells (preCD3CD4%). ROC curves were plotted based on the independent variables (Figure 4a), and their Pearson correlation coefficients with the time of occurrence were −0.1638, 0.1095, 0.2923, 0.1179, 0.0455, and 0.1449, respectively (Figure 4b).

### 3.5. Biomarkers Related to irAEs

**Figure 5:**
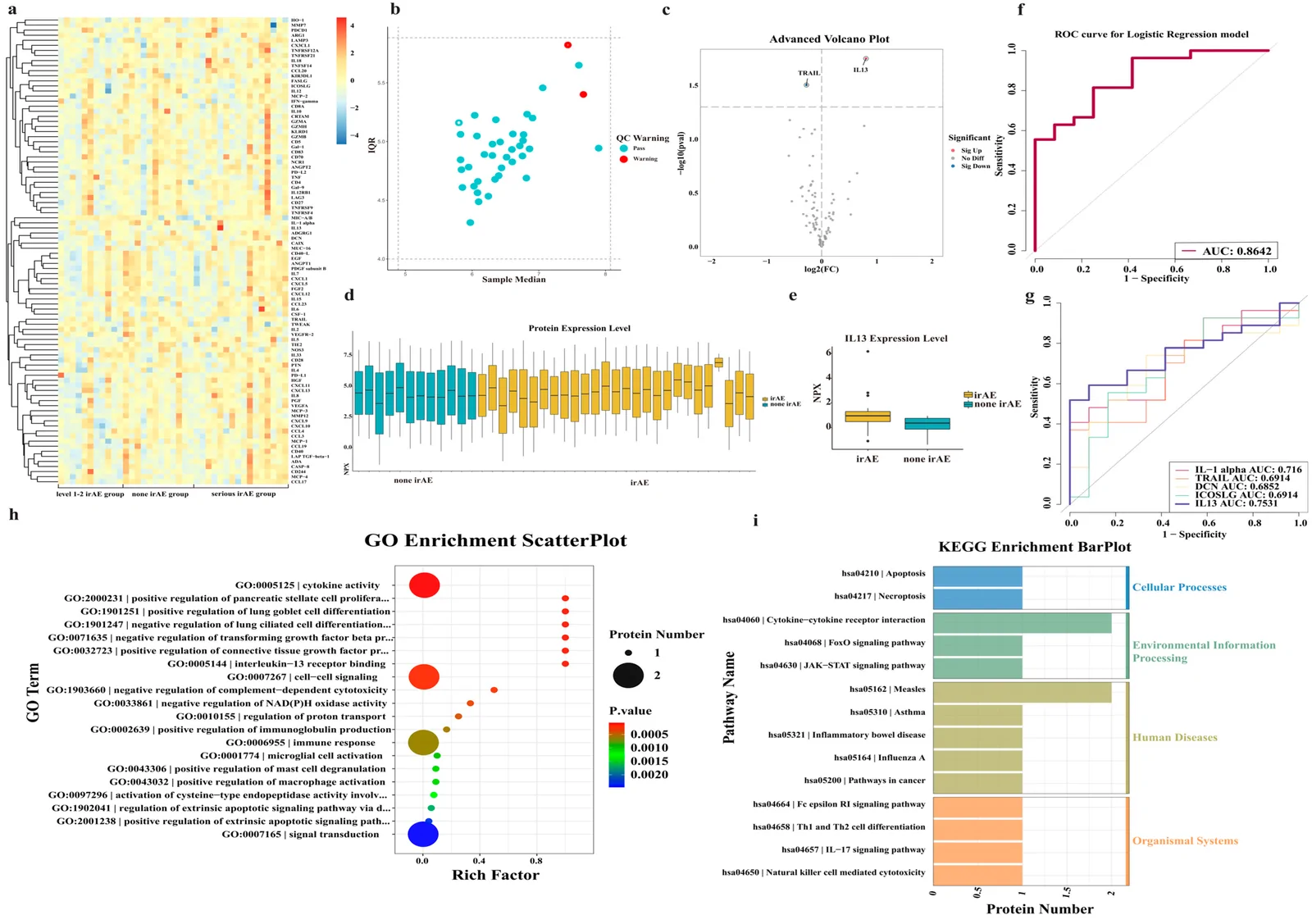
(**a**) Heatmap of protein distribution. (**b**) Quartile range diagram of quality control. (**c**) Volcano plot of differentially expressed proteins between the irAE group and the non-irAE group. (**d**) Box plot of the distribution of sample expression values. (**e**) Distribution difference in IL13 between the two groups. (**f**) The ROC curve of the total top5 proteins in the irAE group vs. the non-irAE group. (**g**) Individual ROC curves of the top5 proteins. (**h**) GO enrichment analysis of top20 differentially expressed proteins in the irAE group vs. the non-irAE group. (**i**) KEGG enrichment analysis of top20 differentially expressed proteins in the irAE group vs. the non-irAE group. Note: (**c**) Red indicates significantly upregulated differentially expressed proteins, blue indicates significantly downregulated differentially expressed proteins, and gray indicates non-differentially expressed proteins; (**e**) IL13 is significantly different between the non-irAE group and the irAE group, and is highly expressed in the irAE group. (**g**) irAE group vs. non-irAE group: the AUCs of IL13 and TRAIL were 0.75 and 0.69, respectively.

Among these 84 patients, we prospectively collected baseline plasma samples (collected within 3 weeks prior to treatment initiation) from a total of 39 cases (27 cases in the irAE group, and 12 cases in the non-irAE group) for Olink Immuno-Oncology panel protein detection (Table 4). The data were sub-grouped, with the irAE group referring to a total of 27 cases from the serious irAE group (*n* = 17) plus the level 1–2 irAE group (*n* = 10). The protein expression distribution is shown in Figure 5a. The internal quality control values of the three sets of samples are relatively uniform (Figure 5b). And the box plot (Figure 5d) shows the distribution statistics of the expression values of each sample, indicating that the samples are reproducible.

Using a *p*-value less than 0.05 obtained from the *t*-test as a screening criterion, proteins meeting this threshold were determined to be differentially expressed. We found that the differentially expressed proteins with statistical significance between the irAE group (27 cases) and the non-irAE group (12 cases) were IL13 and TRAIL (Figure 5c). Specifically, the expression level of IL13 in the non-irAE group was significantly lower than that in the irAE group (Figure 5e). Furthermore, total proteins’ ROC curve and individual ROC curves were plotted for top5 proteins with the smallest *p*-values (Figure 5f,g). Next, we performed GO enrichment analysis, showing the irAE group was mainly enriched in processes such as negative regulation of lung ciliated cell differentiation and positive regulation of lung goblet cell differentiation (Figure 5h). In addition, we performed KEGG pathway analysis, revealing the irAE group was enriched in pathways such as asthma and inflammatory bowel disease (Figure 5i).

### 3.6. Biomarkers Related to Serious irAEs

**Figure 6:**
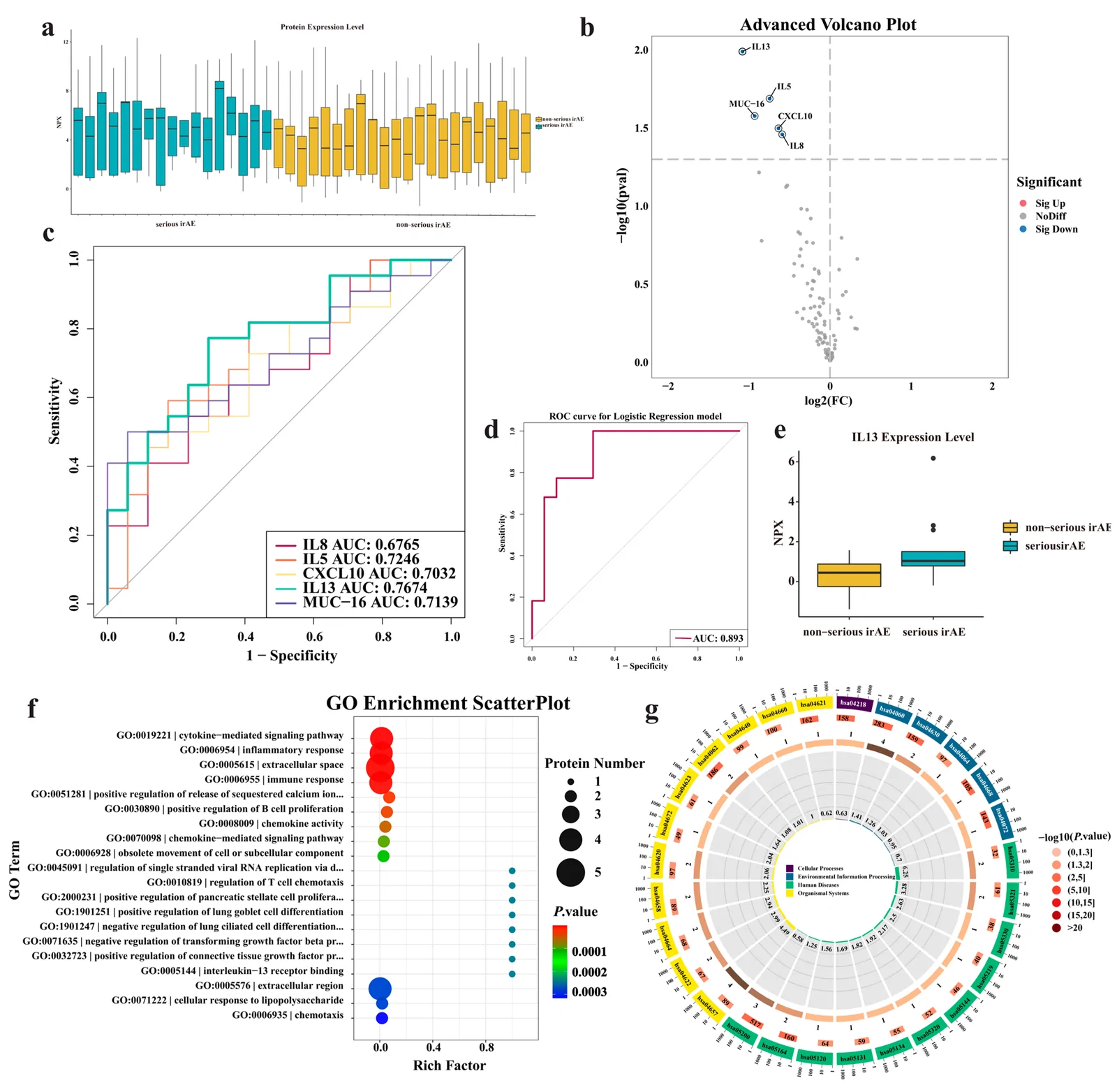
(**a**) Box plot of the distribution of sample expression values. (**b**) Volcano plot of differentially expressed proteins between the serious irAE group and the non-serious irAE group. (**c**) Individual ROC curves of the top5 proteins in the serious irAE group vs. the non-serious irAE group. (**d**) The ROC curve of the total top5 proteins. (**e**) Distribution difference in IL13 between the two groups. (**f**) GO enrichment analysis of top20 differentially expressed proteins in the serious irAE group vs. the non-serious irAE group. (**g**) KEGG enrichment analysis of top20 differentially expressed proteins in the serious irAE group vs. the non-serious irAE group. Note: (**b**) blue indicates significantly downregulated differentially expressed proteins, and gray indicates non-differentially expressed proteins; (**c**) non-serious irAE group vs. serious irAE group: the AUCs of IL13, IL5, MUC-16, CXCL10, and IL8 were 0.77, 0.72, 0.71, 0.70, and 0.68 respectively. (**e**) IL13 is significantly different between the non-serious irAE group and the serious irAE group, and is highly expressed in the serious irAE group.

We divided these 39 cases that underwent Olink Immuno-Oncology panel protein detection into two sub-groups, with the non-serious irAE group referring to a total of 22 cases from the level 1–2 irAE group plus the non-irAE group, and the serious irAE group referring to 17 cases in the serious irAE group (Figure 6a). The differentially expressed proteins with statistical significance between the serious irAE group and the non-serious irAE group were IL13, IL5, MUC-16, CXCL10, and IL8 (Figure 6b). Specifically, the expression level of IL13 in the non-serious irAE group was significantly lower than that in the serious irAE group (Figure 6e). Furthermore, individual ROC curves and total proteins’ ROC curve were plotted for the top5 proteins (Figure 6c,d). Next, we performed GO enrichment analysis, showing the serious irAE group was mainly enriched in processes such as positive regulation of release of sequestered calcium ion intocytosol and positive regulation of B cell proliferation (Figure 6f). In addition, we performed KEGG pathway analysis, revealing the serious irAE group was enriched in pathways such as asthma and IL-17 signaling pathway (Figure 6g).

## 4. Discussion

Through a multi-dimensional integration, this study systematically screened peripheral blood-based biomarkers associated with immune-related adverse events (irAEs). Olink platform-based proteomic analysis revealed that plasma IL13 and TRAIL were significantly correlated with the occurrence of irAEs, while severe irAEs were further associated with IL13, IL5, MUC-16, CXCL10, and IL8. Concurrently, dynamic changes in peripheral lymphocyte subsets, particularly the CD4/CD8 ratio, total T lymphocyte percentage, and NK cell proportion, also displayed strong signals linked to irAEs. These differentially expressed proteins were mainly enriched in biological processes such as negative regulation of lung ciliated cell differentiation and positive regulation of lung goblet cell differentiation, as well as in pathways including asthma and inflammatory bowel disease, which may provide clues for understanding organ-specific toxicity. We also constructed a prediction model incorporating eight variables based on clinical and laboratory indices (AUC = 0.897), which offer more practical candidate tools for early warning and individualized monitoring of irAEs.

Our study found that a decrease in the CD4/CD8 ratio and an increase in total T lymphocytes were associated with the occurrence of irAEs, suggesting a profound remodeling of the T cell compartment following immune checkpoint blockade. The expansion of total T cells reflects systemic immune activation [10,11]. The decline in the CD4/CD8 ratio indicates that this expansion is skewed toward CD8+ cytotoxic T cells, and may be accompanied by a relative insufficiency of immunosuppressive CD4+ regulatory T cells, leading to an effector-dominated pro-inflammatory state and weakened self-tolerance that creates a permissive environment for irAEs [12,13,14,15]. A marked accumulation of highly cytotoxic and proliferative CD8 T cells has been observed in patients with immunotherapy-related colitis [16]. One study identified the regulatory T cell molecule CRTAM as an irAE checkpoint; T cell lineage-specific ablation of Crtam impaired resistance to immune-related toxicity and reduced toxicity in a preclinical tumor-bearing irAE model [17]. Pre-treatment T helper cell count was positively correlated with irAEs. As central coordinators of adaptive immunity, activated T helper cells not only support CD8+ T cell expansion and activation but can also directly contribute to inflammatory injury and even cytokine storm through the secretion of cytokines such as interferon-γ and tumor necrosis factor-α [18,19,20,21,22]. Monitoring the dynamic changes in these cell subsets may help to identify irAEs early, so as to take intervention measures in time.

It is worth mentioning that our research found that IL13 may be a candidate biomarker of irAEs. IL13 is an important immunomodulatory cytokine, mainly secreted by activated Th2 cells, but also produced by mast cells, macrophages, basophils, eosinophils, and B cells [23,24,25,26]. In one observational study, researchers prospectively collected blood samples and, using Luminex-based immunoassays, identified early changes in interleukin family members, including IL-13, that were positively correlated with the occurrence of grade ≥ 2 irAEs [27]. Similarly, another study found that IL-4- and IL-13-related genes were upregulated in immune-related cutaneous adverse events compared with healthy skin [28]. Using the Olink Immuno-Oncology panel to prospectively analyze serial plasma samples from 34 patients with advanced non-small cell lung cancer receiving immune checkpoint inhibitor therapy, researchers observed that elevated baseline levels of IL-4, IL-13, and GZMA after treatment were associated with irAE development, which is highly consistent with our findings [29]. We found that IL13 was enriched in processes such as positive regulation of lung goblet cell differentiation and negative regulation of lung ciliated cell differentiation, as well as in the inflammatory bowel disease pathway, providing molecular clues for the occurrence of pulmonary and intestinal irAEs. The value of these biomarkers lies in helping to guide treatment intensification or deintensification. When patients present with mild, non-specific symptoms, clinicians often find it difficult to determine whether these represent early irAE signals or merely non-specific reactions. In such settings, if IL13 or TRAIL levels are markedly elevated, or the CD4/CD8 ratio drops sharply, this could heighten clinical vigilance, prompting earlier symptomatic management or short-term observation. Conversely, if these markers show no significant changes, clinicians can be more confident in continuing the original treatment regimen, thereby avoiding unnecessary treatment interruptions caused by overinterpretation of non-specific symptoms.

Currently, our research still has some limitations. First, this study is a single-center cohort study with a relatively small sample size, no inclusion of survival outcome data, and a lack of external validation, which may limit the robustness and generalizability of the findings. Olink proteomics analysis was performed on only 39 of the 84 patients, due to some patients declining to sign the supplemental informed consent for plasma storage, and some blood samples not being processed within 2 h of collection, thus precluding their inclusion in the assay. Finally, given the necessity of dynamic surveillance for irAEs, we combined pre-treatment and post-treatment laboratory findings to facilitate a dynamic predictive approach. Nevertheless, it should be acknowledged that the post-treatment variables may not be genuine predictors of irAEs, but instead might be epiphenomena that emerge secondary to the development of irAEs.

In summary, this study suggests that IL13, T helper cells, B cells, and NK cells may serve as potential biomarkers for immune-related adverse events in advanced NSCLC. There are inherent connections between them, and they play an important role in the toxicity of immunotherapy. However, further in-depth research is still needed to further explore its mechanism of action.

## Figures and Tables

**Table 1:** Comparison of general information between irAE group and non-irAE group.

Characteristics	irAE Group (*n* = 57)	Non-irAE Group (*n* = 27)	*p*-Value
Age	67.0 ± 7.5	63.8 ± 10.2	0.11
Height	168.00 [160.00, 170.00]	168.00 [163.00, 172.00]	0.58
Weight	60.00 [49.50, 67.25]	60.00 [55.00, 67.00]	0.47
BMI (kg/m^2^)	22.31 [18.43, 24.97]	21.76 [19.97, 23.70]	0.83
Gender (female)	49 (85.96%)	24 (88.9%)	0.98
PS (0–2)	55 (96.49%)	26 (96.30%)	0.39
PS (3–4)	2 (3.51%)	1 (3.70%)	
Smoking	35 (61.4%)	19 (70.40%)	0.58
Underlying disease			
Hypertension	14 (24.56%)	12 (44.44%)	0.11
Diabetes	8 (14.04%)	4 (14.81%)	1.00
Heart disease	8 (14.04%)	4 (14.81%)	1.00
COPD	8 (14.04%)	1 (3.70%)	0.29
Bronchiectasis	8 (14.04%)	1 (3.70%)	0.29
Hepatitis	1 (1.75%)	3 (11.11%)	0.18
Thyroid nodules	17 (29.82%)	6 (22.22%)	0.64
Clinical staging			0.84
I	2 (3.51%)	1 (3.70%)	
II	8 (14.04%)	4 (14.81%)	
III	14 (24.56%)	9 (33.33%)	
IV	33 (57.89%)	13 (48.15%)	
Pathological classification			0.30
Squamous cell carcinoma	30 (52.63%)	9 (33.33%)	
Adenocarcinoma	22 (38.60%)	16 (59.26%)	
NSCLC-NOS	5 (8.77%)	2 (7.41%)	
Treatment options			0.57
Monotherapy immunotherapy	24 (42.11%)	12 (44.44%)	
Combined with chemotherapy	26 (45.61%)	9 (33.33%)	
Combined with targeted therapy	3 (5.26%)	2 (7.41%)	
Combined with chemotherapy and targeted therapy	4 (7.02%)	4 (14.81%)	
Pre-treatment Laboratory Indicators			
ALT	17.00 [11.00, 27.00]	19.00 [14.50, 24.50]	0.502
TP	66.50 [61.50, 69.40]	67.00 [63.20, 68.55]	0.698
Alb	34.75 (4.04)	37.50 (2.74)	0.002
Cr	67.00 [58.00, 79.00]	78.00 [68.50, 89.00]	0.031
LDH	176.50 [151.25, 226.00]	182.00 [154.00, 209.00]	0.928
Occurrence sites			
Skin	19 (33.33%)		
Incretion	17 (29.82%)		
Liver	8 (14.04%)		
Kidney	3 (5.26%)		
Lung	14 (24.56%)		
Gastrointestinal tract	3 (5.26%)		
Blood system	3 (5.26%)		
Heart	13 (22.81%)		
Nervous system	5 (8.77%)		
Infusion reactions	6 (10.53%)		
Eyes	2 (3.51%)		
Rheumatic system	2 (3.51%)		
Others	5 (8.77%)		

Note: The statistical significance of differences between two groups was assessed using the independent samples t-test for normally distributed continuous variables, the Wilcoxon rank-sum test for non-normally distributed continuous variables, and the Chi-square test (or Fisher’s exact test when appropriate) for categorical variables. Data are presented as n (%) for categorical variables, mean ± SD for normally distributed continuous variables, and median (Q1, Q3) for non-normally distributed continuous variables. Abbreviations: irAE, immune-related adverse event; SD, standard deviation; IQR, interquartile range; BMI, body mass index; PS, performance status; COPD, chronic obstructive pulmonary disease; NSCLC-NOS, non-small cell lung cancer—not otherwise specified; ALT, alanine aminotransferase; TP, total protein; Alb, albumin; Cr, creatinine; LDH, lactate dehydrogenase; NA, not available.

**Table 2:** Significant variables and their coefficients in irAE prediction model.

Significant Variable	Coefficient
preAlb	−0.189242392
preCr	−0.037367351
preCD4/CD8	−0.947756304
postPLT	−0.010862912
postCRP	0.031796982
postLDH	0.012512838
postPNI	−0.044353291
postCD4/CD8	−0.007606856

Note: Coefficient represents the direction and magnitude of the variable’s influence on the outcome variable. A positive coefficient indicates a positive correlation, and a negative coefficient indicates a negative correlation. The larger the absolute value of the coefficient, the greater the influence of the variable on the response variable.

**Table 3:** Significant variables and their coefficients about severe irAEs.

Significant Variable	Coefficient
preBASO	0.0289
preAlb	−0.002
postNEU	0.4512
postTP	−0.2203
postMLR	−0.1924
postPNI	−0.1861
difCD3-CD19+%	−0.1540
difLYM	−0.2223
difNLR	0.0681
difCD3-CD19+	−0.0403

Note: preBASO: pre-treatment basophils, preAlb: pre-treatment albumin, postNEU: post-treatment neutrophils, postTP: post-treatment total protein, postMLR: post-treatment MLR, postPNI: post-treatment PNI, difLYM: difference in lymphocyte, difNLR: difference in NLR, difCD3.CD19: difference in B cells, difCD3.CD19%: difference in B cells percentage.

**Table 4:** Comparison of information between Olink cohort and Not-Olink cohort.

Characteristics	Olink Cohort (*n* = 39)	Not-Olink Cohort (*n* = 45)	*p*-Value
Pathological classification			0.37
Squamous cell carcinoma	21 (53.84%)	18 (40%)	
Adenocarcinoma	16 (41.03%)	22 (48.9%)	
NSCLC-NOS	2 (5.13%)	5 (11.1%)	
Treatment options			0.07
Monotherapy immunotherapy	15 (38.46%)	21 (46.7%)	
Combined with chemotherapy	19 (48.72%)	16 (35.6%)	
Combined with targeted therapy	4 (10.26%)	1 (2.2%)	
Combined with chemotherapy and targeted therapy	1 (2.56%)	7 (15.6%)	
Occurrence sites			
Skin	11 (28.2%)	8 (13.1%)	0.07
Incretion	3 (7.7%)	14 (23%)	0.06
Liver	4 (10.3%)	4 (6.6%)	0.71
Kidney	1 (2.6%)	2 (3.3%)	1.00
Lung	8 (20.5%)	6 (9.8%)	0.15
Gastrointestinal tract	1 (2.6%)	2 (3.3%)	1.00
Blood system	2 (5.1%)	1 (1.6%)	0.56
Heart	2 (5.1%)	11 (18%)	0.07
Nervous system	2 (5.1%)	3 (4.9%)	1.00
Infusion reactions	4 (10.3%)	2 (3.3%)	0.21
Eyes	1 (2.6%)	1 (1.6%)	1.00
Rheumatic system	0 (0%)	2 (3.3%)	0.52
Others	0 (0%)	5 (8.2%)	0.15

Note: For each site row under occurrence sites, perform Fisher’s exact test and adjust for multiple comparisons using the FDR (Benjamini–Hochberg) method.

## Data Availability

The mass spectrometry proteomics data have been deposited to the ProteomeXchange Consortium (https://proteomecentral.proteomexchange.org) via the iProX partner repository [30,31] with the dataset identifier PXD07025, https://proteomecentral.proteomexchange.org/cgi/GetDataset?ID=PXD070025 (accessed on 12 September 2026).

## Abbreviations

**Abbreviations**	**Full Names**
ICIs	immune-checkpoint inhibitors
irAEs	immune-related adverse events
LASSO	least absolute shrinkage and selection operator
PS	performance status scores
COPD	chronic obstructive pulmonary disease
NSCLC	non-small cell lung cancer,
VIF	variance inflation factors
ROC	individual receiver operating characteristic
Tregs	regulatory T cells
TCR	T cell receptor
TME	tumor microenvironment
PMN	Predictive Mean Matching
IQR	interquartile range
BMI	body mass index
WBC	white blood cell count
NEU	neutrophil count
LYM	lymphocyte count
MONO	monocyte count
BASO	basophil count
HB	hemoglobin
TP	total protein
Alb	albumin
PNI	prognostic nutritional index
SII	systemic immune inflammation index
